# DNA Methyl Transferase (DNMT) Gene Polymorphisms Could Be a Primary Event in Epigenetic Susceptibility to Schizophrenia

**DOI:** 10.1371/journal.pone.0098182

**Published:** 2014-05-23

**Authors:** Koramannil Radha Saradalekshmi, Nanoth Vellichiramal Neetha, Sanish Sathyan, Indu V. Nair, Chandrasekharan M. Nair, Moinak Banerjee

**Affiliations:** 1 Human Molecular Genetics Laboratory, Rajiv Gandhi Centre for Biotechnology, Thiruvananthapuram, Kerala, India; 2 Mental Health Centre, Thiruvananthapuram, Kerala, India; 3 Nair's Hospital, Ernakulam, Kerala, India; University of Adelaide, Australia

## Abstract

DNA methylation has been implicated in the etiopathology of various complex disorders. DNA methyltransferases are involved in maintaining and establishing new methylation patterns. The aim of the present study was to investigate the inherent genetic variations within DNA methyltransferase genes in predisposing to susceptibility to schizophrenia. We screened for polymorphisms in DNA methyltransferases, *DNMT1*, *DNMT3A*, *DNMT3B* and *DNMT3L* in 330 schizophrenia patients and 302 healthy controls for association with Schizophrenia in south Indian population. These polymorphisms were also tested for subgroup analysis with patient's gender, age of onset and family history. *DNMT1* rs2114724 (genotype P = .004, allele P = 0.022) and rs2228611 (genotype P = 0.004, allele P = 0.022) were found to be significantly associated at genotypic and allelic level with Schizophrenia in South Indian population. *DNMT3B* rs2424932 genotype (P = 0.023) and allele (P = 0.0063) increased the risk of developing schizophrenia in males but not in females. *DNMT3B* rs1569686 (genotype P = 0.027, allele P = 0.033) was found to be associated with early onset of schizophrenia and also with family history and early onset (genotype P = 0.009). *DNMT3L* rs2070565 (genotype P = 0.007, allele P = 0.0026) confers an increased risk of developing schizophrenia at an early age in individuals with family history. In-silico prediction indicated functional relevance of these SNPs in regulating the gene. These observations might be crucial in addressing and understanding the genetic control of methylation level differences from ethnic viewpoint. Functional significance of genotype variations within the DNMTs indeed suggest that the genetic nature of methyltransferases should be considered while addressing epigenetic events mediated by methylation in Schizophrenia.

## Introduction

Schizophrenia is one of the most debilitating mental disorders affecting 1% of the world population. Being a complex disorder with heterogeneous nature of symptom presentations the etiology of schizophrenia is still an enigma. The disorder has a well established heritability, while studies done in identifying the genetic susceptibility factors were invariantly inconsistent. Genome wide association studies and Linkage analyses have tried to identify susceptibility loci but, no common genetic variant confers in itself more than twice the risk in susceptibility for schizophrenia in general population [1]. Apart from genetic factors, several environmental factors have been implicated in the etiology of schizophrenia which includes migration [2], urbanicity during upbringing [3], prenatal famine [4], season of birth [5], Paternal age [6], in utero exposure to influenza epidemics [7], cannabis use [8] etc. Several studies have been done worldwide to elucidate the role of Gene X Environment interaction in vulnerability to schizophrenia [9]. DNA sequence variations, epigenetic regulations and environmental cues act stochastically to contribute in the etiopathogenecity of schizophrenia [10]. Recent advances in the field of epigenetics have increased understanding of this interaction by identifying molecular mechanisms that mediate environmental influences on gene expression and activity. Epigenetic mis - regulations in response to a variety of environmental factors have been suggested as a mechanism to explain the increasing risk of schizophrenia in adulthood [11]. Epigenetic mechanisms represent a form of cellular memory that contributes to either short- or long-term changes in neuronal function in response to a variety of behavioral experiences, environmental factors, and pharmacological stimuli [12].

Epigenetic changes are mediated by chemical modification of DNA such as DNA methylation or by protein modifications such as histone acetylation and methylation. DNA methylation is indeed the most studied and probably the best understood type of epigenetic modification. DNA methylation is a covalent modification occurring at the cytosine residues of DNA with lasting heritable effects. DNA methylation usually at the CpG sites near to the regulatory regions of genes and regulate the transcription of these genes. DNA methylation at the gene promoters can affect gene transcription by altering the accessibility of RNA polymerase and transcription factors [13]. Methylation of DNA typically leads to transcriptional repression. The process of DNA methylation is regulated by several external and internal factors which include age, diet, folate levels, methionine turn over and the molecular level maintenance machinery. DNA Methyltransferases are a family of enzymes that mediate the process of DNA methylation as a significant component of molecular level maintenance machinery. DNMTs catalyses the transfer of methyl group from S-Adenosyl methionine to the cytosine residue of DNA. There are mainly two classes of DNA methyltransferases; maintenance methyltransferases (DNMT1) and de novo methyltransferases (DNMT3A & 3B). DNMT1 has a preference for hemi methylated DNA and helps in maintaining the methylation pattern through generations. DNMT3A and 3B induces de novo methylation to establish tissue specific DNA methylation pattern during development and in response to environmental factors. DNMT3L lacks methyltransferase activity but orchestrates the process of methylation by the de novo methyltransferases. It also interacts with other DNA binding proteins in chromatin remodeling complex. Genetic variants which influence the DNA methylation need to be considered given the importance of this in regulation of this epigenetic process in response to the environmental cues. The aim of the present study was to identify the role of genetic variants in DNA MethylTransferases such as maintenance methyltransferase gene (*DNMT1*) and genes for de novo methyltransferases (*DNMT3A* & *3B*) and *DNMT3L* in predisposing to schizophrenia. The role of these variants would further be investigated and evaluated in terms of age of onset, gender and family history.

## Materials and Methods

### Subject selection

Patients in this study were recruited from various Mental Health Centres across Kerala, a South Indian state. The study group consisted of 330 patients and 302 controls. All the patients and controls belong to Malayalam speaking ethnic communities reflecting their genetic stratification. Linguistic ethnic background of Malayalam speaking ethnic communities from Kerala also indicates that the population was genetically and epigenetically stratified by considering the food, environment and regional parameters. Patients diagnosed with schizophrenia were recruited by psychiatrists after evaluation for symptoms using ICD10/DSM IV criteria and were rated using BPRS-E for symptom severity. Demographic details regarding age, ethnicity, family history, diet etc was collected through personal interviews during sample collection. Age of onset was determined as the time when the first schizophrenia symptoms occurred based on interview with patient and family informants or from medical records. Age of onset ≤18 years was considered as early onset and was compared for association against the normal and late onset patients. 10 ml peripheral blood sample was collected in EDTA vials, for DNA isolation, from the study subjects. Genomic DNA was isolated from peripheral lymphocytes using conventional Phenol Chloroform method. All the participants gave informed, written consent in a standard consent form to participate in the study after being provided with, and receiving a full explanation of study protocols and objectives. All potential participants who declined to participate or otherwise did not participate were eligible for treatment (if applicable) and were not disadvantaged in any other way by not participating in the study. Whenever the patient had a compromised capability to consent next of kin or legally authorised representative had consented on the behalf of participants. The present study was approved by the Institutional Ethics Committee for Biomedical Subject of Rajiv Gandhi Center for Biotechnology, as per the guidelines of Indian Council for Medical Research and was also approved by Directorate of Health Services, Govt. of Kerala. Age, Sex and ethnicity matched controls were recruited from the normal population.

### SNP selection and Genotyping

SNPs were selected based on functional significance, minor allele frequency and their tagging status. Eight SNPs from *DNMT1*, four from *DNMT3A*, three from *DNMT3B* and four from *DNMT3L* were selected. The details of the selected SNPs are given in **Table 1**. Genotyping was done using fluorescence-based competitive allele-specific PCR, KASPar (KBiosciences LGC, Herts, UK) as per the manufacturer's protocol and confirmed by sequencing in representative samples. The genotype calling based on the respective allele specific fluorescence was done by allelic discrimination utility of the SDS 7500 v2.0.5 software (Applied Biosystems, Foster City, CA, USA) at an ambient temperature of 25°C and genotype clusters were plotted.

**Table 1:** Details of Seleted SNPs.

Gene	SNP ID	Alleles	Genotyping Method	Possible Functional Effects
***DNMT1***	rs10418707	G/A	KASPar	Intronic enhancer
	rs2114724	C/T	KASPar	Intronic enhancer
	rs2228611	G/A	KASPar	Splicing Regulation
	rs10423341	C/A	KASPar	Intronic enhancer
	rs2228612	A/G	KASPar	Missense (conservative)
	rs2162560	G/A	KASPar	Intronic enhancer
	rs759920	G/A	KASPar	Intronic enhancer
	rs16999593	T/C	KASPar	Missense (non-conservative); Splicing regulation
***DNMT3A***	rs1550117	G/A	Sequencing	
	rs2304429	G/A	KASPar	Intronic enhancer
	rs2289195	G/A	KASPar	
	rs734693	C/T	KASPar	Intronic enhancer
***DNMT3B***	rs1569686	T/G	KASPar	Promoter/regulatory region
	rs2424913	C/T	KASPar	
	rs2424932	G/A	KASPar	
***DNMT3L***	rs8129776	G/A	KASPar	
	rs762424	G/A	KASPar	Intronic enhancer
	rs2070565	C/T	KASPar	Splicing site
	rs2838535	C/T	KASPar	

### Statistical Analysis

Genotype and allele frequencies were computed and were checked for deviation from hardy Hardy-Weinberg equilibrium (ihg2.helmholtz-muenchen.de/cgi-bin/hw/hwa1.pl). Case-control genetic comparisons were performed using the chi-square test and allelic and model based odds ratios (OR), and 95% confidence intervals (CI) were calculated by Fisher's exact test (two-tailed). Further stratification of the patients was done to understand the role of *DNMT* variants with the gender, age of onset and family history. All statistical analyses were performed using the Graph Pad Prism 5.01, (GraphPad software Inc. San Diego, CA, USA). We considered p-value of <0.05 as significant. We carried out Bonferroni's correction to test for multiple comparisons. However due to the exploratory nature of this study, we prefer to present the results with no adjustment for multiple testing as well, so as not to penalize the data with the possibility of missing important findings. Haplotype analysis was performed using unphased 3.1.3 and P values were visualized using a Microsoft excel based graphical tool GrASP v0.82 beta. Linkage Disequilibrium plots for controls and patients were generated using Haploview 4.2. LD plots for different world populations were generated for the selected SNPs (snpinfo.niehs.nih.gov/snpinfo/snptag.htm). Functional prediction of the deleterious effect if any, of the associated SNP with respect to the functional categories such as protein coding, splicing regulation, transcriptional regulation, and post-translation was assessed in silico using F-SNP program (compbio.cs.queensu.ca/F-SNP/), FastSNP (fastsnp.ibms.sinica.edu.tw), SNP Function Prediction (FuncPred) (snpinfo.niehs.nih.gov/snpinfo/snpfunc.htm), SNP Nexus (www.snp-nexus.org/), HaploReg (www.broadinstitute.org/mammals/haploreg).

## Results

**Figure 1:**
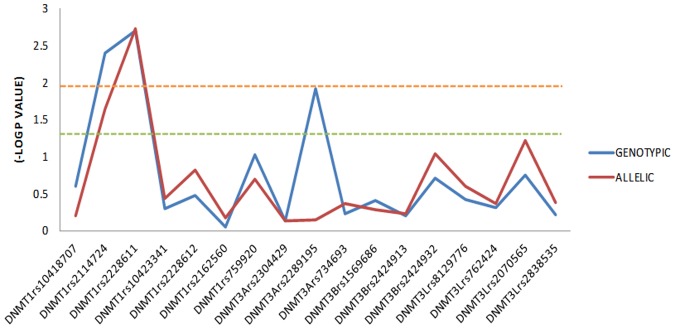
Association analysis of *DNMT1, DNMT3A, DNMT3B* and *DNMT3L* polymorphisms represented in -Log P value with schizophrenia in a South Indian population.

**Figure 2:**
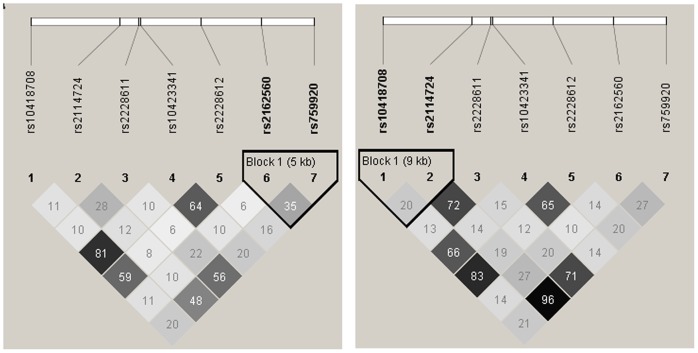
Linkage Disequilibrium plot showing R″ value in patients (A) and controls (B).

The demographic characteristics of the patients enrolled in this study are shown in **Table 2**. There were no significant differences in the mean age and sex distribution between patients and controls. There was no evidence of a deviation from Hardy-Weinberg equilibrium among the controls (P>0.05). Observations from association analyses of SNPs in *DNMT1, DNMT3A, DNMT3B*, and *DNMT3L* indicated that *DNMT1* rs2114724 and rs2228611 were strongly associated with schizophrenia at allelic and genotypic level (**Table 3** **, Table S1**). Subgroup analysis of *DNMT* polymorphisms within the patient based on Gender, Age of onset and Family history is shown in **Table 4**. Association analysis of *DNMT1, DNMT3A, DNMT3B* and *DNMT3L* polymorphisms are summarised by -Log P value for schizophrenia in a South Indian population (**Figure 1**). In *DNMT1* rs2114724 the allelic (P = 0.022, OR 1.3, CI = 1.04–1.62) and genotypic (P = 0.004) association show an overrepresentation of TT genotype and T allele while in *DNMT1* rs2228611 the allelic (P = 0.002 OR 1.43, CI = 1.14–1.8) and genotypic (P = 0.003) association show and overrepresentation of AA genotype and A allele in schizophrenia patients (**Table 3**). In-Silico analysis of *DNMT1* rs2114724 and rs2228611 SNPs, indicate highest functional significance within the *DNMT1* gene. *DNMT1* rs2114724 indicated enhancer like function with F score of 0.21 while rs2228611 indicated functional relevance for splicing regulation with an F-score of 0.31 (**Table 5**). While evaluating the allele and genotype frequency differences of the associated *DNMT1* SNPs with HapMap populations, we do observe variations within the Indian population i.e between Gujarati Indian (GIH) and South Indian (KER) population. The minor allele of *DNMT1* rs2228611 in South Indian and Han Chinese (CHB) populations turns out to be a major allele in Mexican and CEU population (**Figure S1**) while the minor allele *DNMT1* rs2114724 in south Indian population turns out to be a major allele in CHB population (**Figure S2**). Linkage disequilibrium analysis revealed distinct differences in the LD pattern between patients and controls of *DNMT1* due to the associated SNPs (**Figure 2**).

**Table 2:** Demographic characteristics of Patients.

Cases	Total	330
	Females	196
	Males	134
Mean Age,	Females	36.77±11.65
	Males	34.85±8.93
Mean Age of onset	Females	25.3±9.06
	Males	24.03±7.47
Positive Family History		41%
Age of onset ≤18 years		27%

**Table 3:** Association analysis of *DNMT1* polymorphisms in schizophrenia.

SNP	Cohort		Genotypes		P value	Allele		P value	Odds Ratio	95% C.I
		**G/G**	**A/G**	**A/A**		**G**	**A**			
*DNMT1*	PATIENTS	179(0.58)	111(0.36)	18(0.06)	0.249	469(0.76)	147(0.24)	0.6388	1.068	0.8212–1.390
rs10418707	CONTROLS	159(0.53)	126(0.42)	13(0.04)		439(0.75)	147(0.25)			
		**C/C**	**C/T**	**T/T**		**C**	**T**			
*DNMT1*	PATIENTS	97(0.30)	147(0.45)	81(0.25)	**0.004**	347(0.53)	311(0.47)	**0.0223**	1.3	1.04–1.62
rs2114724	CONTROLS	98(0.33)	160(0.53)	43(0.14)		356(0.59)	246(0.41)			
		**G/G**	**A/G**	**A/A**		**G**	**A**			
*DNMT1*	PATIENTS	97(0.30)	155(0.48)	68(0.21)	**0.003**	349(0.55)	291(0.45)	**0.0019**	1.43	1.14–1.80
rs2228611	CONTROLS	113(0.38)	152(0.51)	34(0.11)		378(0.63)	220(0.37)			
		**C/C**	**A/C**	**A/A**		**C**	**A**			
*DNMT1*	PATIENTS	185(0.59)	112(0.36)	17(0.05)	0.512	482(0.78)	134(0.22)	0.3623	0.88	0.67–1.16
rs10423341	CONTROLS	163(0.56)	115(0.40)	12(0.04)		441(0.76)	139(0.24)			
		**A/A**	**A/G**	**G/G**		**A**	**G**			
*DNMT1*	PATIENTS	182(0.59)	112(0.36)	14(0.05)	0.33	476(0.77)	140(0.23)	0.1505	0.82	0.63–1.07
rs2228612	CONTROLS	153(0.54)	111(0.39)	19(0.07)		417(0.74)	149(0.26)			
		**G/G**	**A/G**	**A/A**		**G**	**A**			
*DNMT1*	PATIENTS	156(0.49)	130(0.41)	33(0.10)	0.905	442(0.69)	196(0.31)	0.6659	0.95	0.74–1.21
rs2162560	CONTROLS	139(0.47)	124(0.42)	32(0.11)		402(0.68)	188(0.32)			
		**G/G**	**A/G**	**A/A**		**G**	**A**			
*DNMT1*	PATIENTS	103(0.32)	153(0.47)	68(0.21)	0.096	359(0.55)	289(0.45)	0.2069	1.16	0.92–1.45
rs759920	CONTROLS	96(0.32)	157(0.53)	43(0.15)		349(0.59)	243(0.41)			

**Table 4:** Association analysis based on gender, Age of onset and Family history.

Demographic variables	SNP ID	Gene	Associated genotype/Allele	Genotype P Value	Allele P Value
Male Patients		*DNMT3B*	GG/G	0.023	0.0063
Age of Onset below 18	rs1569686	*DNMT3B*	TT/T	0.027	0.033
Family history + Age of Onset below 18	rs1569686	*DNMT3B*	TT/T	0.009	0.07
Family history + Age of Onset below 18	rs2070565	*DNMT3L*	CC/C	0.007	0.0026

**Table 5:** In-Silico functional analysis of associated SNP of DNMTs in schizophrenia.

SNP	F Score	Functional role	Binding Affinity scores			Source
rs2114724	0.208	Regulatory motifs altered	**Motif**	T	C	Haploreg
			ETF	−0.4	11.3	
rs2228611	0.314	Regulatory motifs altered	Motif	A	G	Haploreg
			Bach1	6.9	−5	
			Bach2	11.8	−0.1	
			ROα1_3	12.3	0.4	
		Splice Regulation	**Exon splicing enhancer**	A	G	ESEfind
			SRp55	3.08	-	
			SF2ASF1	-	4.03	
rs1569686	-	Regulatory motifs altered	**Motif**	G	T	Haploreg
			GR_disc5	12.1	3.1	
			Gfil_1	0.8	11.6	
			Gfil_2	9.1	11.6	
			Gfilb	9.2	11.5	
			LBP-1_2	−0.4	11.6	
			NF-kappaB_disc3	14.2	10	
rs2424932	-	miRNA Binding	**miRNA**	A	G	SNP FuncPred
			hsa-miR-920	140	-	
			hsa-miR-4802-5p	152	-	mirSNP
rs2070565	1.00	Regulatory motifs altered	**Motif**	T	C	Haploreg
			ATF4	2.2	13.7	
			Maf_known1	13.1	3.8	
			NF-E2_disc1	10.7	10.6	
			Nrf-2_2	16.2	6.5	
			nrf-2_3	13.9	11.3	

Among de novo methyltransferases the *DNMT3A* rs2289195 was found to be associated at genotypic level (P = 0.013) with schizophrenia. None of the selected SNPs in *DNMT3B*, and *DNMT3L* were found to be associated with schizophrenia. We further analysed the association of *DNMT* polymorphisms within the patient group by classifying the patients into different subgroups based on Gender, Age of onset and Family history (**Table 4**). *DNMT3B* rs2424932 GG genotype (P = 0.023) and G allele (P = 0.0063) was found to be associated with the Schizophrenia in male patients when compared against male controls. Similar association was not observed in females. In the subgroup analysis with age of onset and family history we found that *DNMT3B* rs1569686 was associated with genotypic (P = 0.027) and allelic (P = 0.033) level with an overrepresentation of TT genotype and T allele in patients with early onset of Schizophrenia. Patients with positive family history of psychiatric illness were further subgrouped into early onset and late onset. Interestingly here too *DNMT3B* rs1569686 was also found to be associated significantly at genotypic level (P = 0.009) with family history and early age of onset. rs1569686 (−579 G/T) is a promoter SNP inducing changes in Transcription factor binding affinity (mbs.cbrc.jp/research/db/ TFSEARCH.html). Further, the subgroup analysis of positive family history and early onset and normal or late onset indicated that *DNMT3L* rs2070565 was significantly associated at genotypic (P = 0.007) and allelic (P = 0.0026) level with over representation of CC genotype and C allele in early onset subgroup (**Table 4**). In-silico analysis indicated that *DNMT3L* rs2070565 is a splice site variant with an F-score of 1. rs2070565 T/C is an intronic SNP in *DNMT3L* which result in alteration of splice regulatory motifs (**Table 5**).

## Discussion

In the present study we report that the polymorphisms in the *DNMT1*, rs2114724 and rs2228611 are significantly associated with schizophrenia in Malayalam speaking south Indian population. Subsequently, while stratifying the Schizophrenia patient group based on the demographic variables, we report a strong association with de novo *DNMTs*. Among the de novo *DNMTs* in the present study, *DNMT3B* rs2424932 was strongly associated with gender and *DNMT3B* rs1569686 early age of onset while *DNMT3L* rs2070565 was found to be associated with family history and early age of onset. *DNMT1* is known to be responsible in maintaining the intrinsic methylation machinery through generations while de novo DNMTs induces de novo methylation to establish tissue specific DNA methylation pattern during development and in response to environmental factors. These observations suggest that while addressing the alterations in methylation events contributing to schizophrenia, it is important to evaluate the underlying genetic variants in methyltransferases to understand the reasons for differential methylation.

Till date most of the studies relating to schizophrenia has been restricted to either expression based analysis or methylation analysis at candidate gene or global methylation level changes. Hypomethylation of leukocyte DNA in male subjects with schizophrenia was reported in Japanese population [14]. Interestingly, in another study on Israel population no differences in global methylation status was observed inspite of an increase in homocysteine levels in schizophrenia patients [15]. Gene specific methylation and subsequent alteration in their expression level has been studied with many candidate genes in different populations. Methylation in *RELN, SOX10, MB-COMT, 5HTR, DRD2* etc. has been studied extensively. The hypermethylation of the CpG islands flanking a CRE and SP1 binding site observed at upstream to Reelin promoter in the post-mortem brains of schizophrenic patients [16]–[17]. *MB*-*COMT* promoter DNA has been reported to be hypomethylated in schizophrenia and bipolar disorder patients, compared with the controls [18]. However, these findings could not be confirmed when replicated in other studies [19]–[21]. Increased DNA methylation in the promoter region of the *5HTR1A* gene has also been reported in SCZ and BPD [22]. However, no significant difference in the overall DNA methylation of *MAOA* promoter was reported in schizophrenia patients [23]. In cortical Brodmann's area 10 (BA10) and peripheral blood lymphocytes of Schizophrenia patients, *DNMT1* and to a lesser extent *DNMT3a*, mRNAs were reported to be upregulated [24].The increased expression of S-adenosyl methionine and DNA methyltransferase-1 has been postulated to contribute to promoter cytosine 5-methylation and to downregulation of the expression of mRNAs encoding for reelin and GAD67 in cortical GABAergic neurons of schizophrenia and bipolar disorder patients [25]. Abberant gene expression of brain-derived neurotrophic factor (BDNF) has been implicated in onset of several mental illnesses subsequent to early-life adversity. Maltreatment during infancy leads to increased methylation in *BDNF* promoter and a subsequent decrease in its expression. [26] Interestingly, all these studies refer to alterations in methylation levels as an influence by the extrinsic factors such as environment, food intake etc. As a result, the success of these observations on methylation levels or expression levels of candidate genes has been limited to individual studies which could not be replicated in many other studies as number of extrinsic and intrinsic factors could modulate the outcome. None of these studies have investigated the role of intrinsic mechanisms in maintaining the methylation machinery to recognize the inherent genetic factors that are crucial to influence the methylation levels in disease causation. The present study on the role of polymorphisms in DNMTs could help in understanding the discrepancies of inconsistencies on differential methylation in previous studies.

In the present study the *DNMT1* rs2114724 TT genotype and T allele and *DNMT1* rs2228611 AA genotype and A allele were observed to be significantly associated with schizophrenia. These observations are interesting as *DNMT1* rs2114724 is an intronic SNP with mutant allele T rendering an intronic enhancer effect. In Korean population, this *DNMT1* rs2114724 has been reported to be associated with HBV clearance through intronic enhancer effect [27]. LINE-1 methylation which is a surrogate marker for global methylation was reported to be higher in men with atleast one mutant allele compared to wild type genotype [28]. Moreover the positive correlation of LINE-1 methylation with S-Adenosyl methionine which was seen in individuals with wildtype genotype was lost in men with heterozygous and mutant genotypes [28]. Similarly, *DNMT1* rs2228611 which is a synonymous SNP found in exon 17 and was found to be associated with schizophrenia in our study, has a possible splice regulatory function with G/A resulting in loss of three Exonic splicing enhancer binding motif (see Supplementary data). AG and AA genotype of *DNMT1* rs2228611 SNP has been reported to be associated with strong inverse relation of LINE-1 methylation with Cadmium exposure in Argentinean women [29] suggesting a possible role in regulating the methylation level in response to environmental cues. Functional significance of genotype variations within the *DNMT*s indeed suggest that the genetic nature of methyltransferases should be considered while addressing the issue of methylation in Schizophrenia.

The intrinsic mechanisms such as age, gender, ethnicity and genotype and extrinsic mechanisms such as food and environment, if not understood in relation to each other, can also drastically influence inferences on epigenomic alterations. Epigenomic changes are strongly influenced by age, gender, ethnicity and environment. None of the earlier studies on schizophrenia have discussed on these variables. In the present study there were no significant differences in the mean age and sex distribution between patients and controls, indicating that the variables such as age and gender did not contribute to epigenomic alteration. Subject selection based on Malayalam speaking ethnicity also indicated that the populations were genetically stratified. In an earlier study we have reported that Malayalam speaking ethnic communities are genetically distinct from the north Indian communities [30]. While comparing the associated allele and genotype distributions of *DNMT1* with the HapMap population we do observe distinct variations within the Indian population i.e Gujarati Indian and South Indian population. Even while comparing with the global populations we observe that the minor allele of *DNMT1* rs2228611 in South Indian and CHB populations turns out to be a major allele in Mexican and CEU population while the minor allele *DNMT1* rs2114724 in south Indian population turns out to be a major allele in CHB population. In India it is also possible to generalize the epigenetic parameters based on linguistic background of the ethnic population as all linguistic background have distinct food habits which is different from each linguistic ethnic population. In the present study the since the sampling was done from within the Malayalam speaking ethnic community from Kerala which also indicates that the samples were epigenetically stratified as they have similar life style based on their food habits. These observations indicate that genetic selection based on ethnicity might have differential impact on genetic association and epigenomic studies significantly.

Stratifying the patients based on their genotypes and demographic variables we observe a strong association of *DNMT3B* rs2424932 GG genotype and G allele with schizophrenia in male patients. Interestingly, the *DNMT3B* rs2424932 A allele was recently reported to be associated with suicidal tendency in people with psychiatric illness along with increase in global DNA methylation level in the suicide attempters compared to non attempters [31]. This SNP is found in 3′UTR and is present in miRNA binding site. Insilico analyses revealed that presence of A allele adds an additional regulation to the gene through binding of hsa_mir-920. In our study, we report an association of *DNMT3B* rs1569686 TT genotype and T allele with early onset of schizophrenia. In-silico analysis revealed that this *DNMT3B* rs1569686 (−579 G/T) is a promoter SNP inducing changes in Transcription factor binding affinity (mbs.cbrc.jp/research/db/ TFSEARCH.html). Earlier reports do indicate that null mutations of the mammalian DNA methyltransferases (*DNMT*s) can be lethal either at prenatal or and postnatal stage [32]–[33]. The exact functional role of the *DNMT3B* rs1569686 (−579 G/T) polymorphism is still not yet completely elucidated. The *DNMT3B* has nearly 40 known splice variants expressed in a tissue- and disease-specific manner, but very little is known about the role of these splice variants in modulating DNMT3B function. The polymorphic regions −149C>T, −283T>C and −579G>T within the *DNMT3B* gene has two transcriptional start sites, which exist in different exons (exon 1A and 1B) and the expression is regulated by different promoters. One promoter is nested within a CpG-rich area, whereas the other promoter is found in CpG poor. The *DNMT3B* - rs6058870 (283T>C, −283 bp from exon 1A transcription start site) and rs1569686 (−579G>T, −579 bp from exon 1B transcription site) polymorphisms are located in the CpG-rich and CpG-poor promoters, respectively. Some authors have suggested rs1569686 (−579G>T) might directly impair promoter activity while majority observe that it might induce its effect through a tag SNP of functional haplotypes with −149C>T (rs2424913) and 283T>C (rs6058870), which have been functionally associated with promoter activity and gene expression levels. It is difficult to synthesize a construct of a haplotype containing −149C>T, −283T>C and −579G>T polymorphisms by PCR since the −579G>T polymorphism is located at 17171 bp from the −283T>C polymorphism. This makes haplotype specific functional observation difficult. A recent study has demonstrated the potential of combining functional genomics and population genetics approaches for understanding gene regulation (**Spivakov et al. 2012**). Therefore, while evaluating the LD patterns of SNPs and SNPs proximal to the studied SNPs, among different HapMap population we observe distinct differences between various ethnicities. LD patterns of GIH population show distinct differences with African and Mongoloid populations. *DNMT3L* rs2070565 CC genotype and C allele was found to be significantly higher in patients with family history of mental illness and who developed the disease at an earlier age compared to those who developed at normal or older age. This suggests the role of *DNMT3L* rs2070565, in predisposing individuals with a positive family history with increased risk of developing Schizophrenia at an early age. This could be mediated through an alteration in splice regulatory motifs.

To date this is the first exhaustive study implicating the role of *DNMT1* with Schizophrenia, *DNMT3B* with male gender and early onset and *DNMT3L* rs2070565 with early onset linked to positive family history patients. Implicating the role of *DNMT1* polymorphisms that are crucial in maintenance of an already established DNA methylation patterns at replication and de novo *DNMT3A, 3B* and *3L* that are crucial in establishing new methylation patterns is indeed a step forward towards understanding methylation level differences in Schizophrenia. These observations might be crucial in addressing and understanding the genetic control of methylation level differences from ethnic viewpoint. It would be interesting to identify the implication of these observations in global methylation, genome wide methylation and schizophrenia candidate gene specific methylation.

## Supporting Information

Figure S1 **Allele (A) and genotype (B) frequency of** ***DNMT1*** **rs2228611 in HapMap population in comparison to South Indian population (KER).**
(TIF)

Figure S2 **Allele (A) and genotype (B) frequency of** ***DNMT1*** **rs2114724 in HapMap population in comparison to South Indian population (KER).**
(TIF)

Table S1 **Association analysis of** ***DNMT3A, DNMT3B*** **and** ***DNMT3L*** **polymorphisms in schizophrenia.**
(DOC)
