# Supplementary figures and images for: DNA Methyl Transferase (DNMT) Gene Polymorphisms Could Be a Primary Event in Epigenetic Susceptibility to Schizophrenia
Source: PLoS One. 2014 May 23;9(5):e98182. doi: 10.1371/journal.pone.0098182 (PMC4032286; doi:10.1371/journal.pone.0098182)

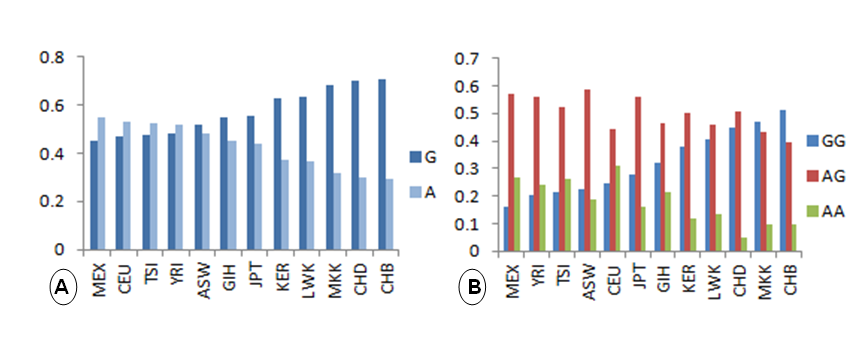

Supplement: Figure S1 — Allele (A) and genotype (B) frequency of DNMT1 rs2228611 in HapMap population in comparison to South Indian population (KER). (TIF) [file pone.0098182.s001.tif]

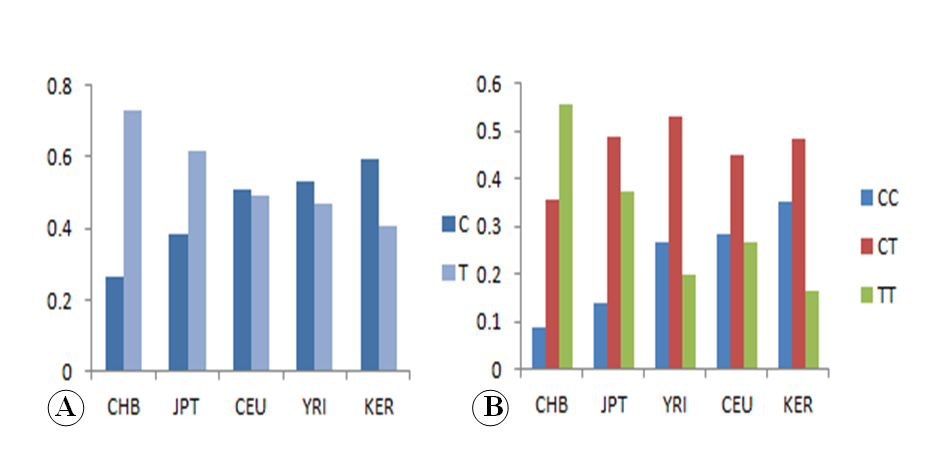

Supplement: Figure S2 — Allele (A) and genotype (B) frequency of DNMT1 rs2114724 in HapMap population in comparison to South Indian population (KER). (TIF) [file pone.0098182.s002.tif]
