# Supplementary material for: DNA Methyl Transferase (DNMT) Gene Polymorphisms Could Be a Primary Event in Epigenetic Susceptibility to Schizophrenia
Source: PLoS One. 2014 May 23;9(5):e98182. doi: 10.1371/journal.pone.0098182 (PMC4032286; doi:10.1371/journal.pone.0098182)
Supplement: Table S1 — Association analysis of DNMT3A, DNMT3B and DNMT3L polymorphisms in schizophrenia. (DOC) [file pone.0098182.s003.doc]

**Supplementary Table 1: Association analysis of DNMT3A, DNMT3B and DNMT3L polymorphisms in schizophrenia**

| **SNP** | **Cohort** |  | **Genotypes** |  | **P value** | **Allele** |  | **P value** | **Odds Ratio** | **95% C.I** |
| --- | --- | --- | --- | --- | --- | --- | --- | --- | --- | --- |
|  |  | **G/G** | **A/G** | **A/A** |  | **G** | **A** |  |  |  |
| DNMT3A | PATIENTS | 109(0.34) | 158(0.49) | 57(0.18) | 0.739 | 376(0.58) | 272(0.42) | 0.727 | 0.96 | 0.77 - 1.20 |
| rs2304429 | CONTROLS | 101(0.34) | 138(0.46) | 59(0.20) |  | 340(0.57) | 256(0.43) |  |  |  |
|  |  | **G/G** | **A/G** | **A/A** |  | **G** | **A** |  |  |  |
| DNMT3A | PATIENTS | 145(0.45) | 155(0.48) | 22(0.07) | 0.013 | 445(0.69) | 199(0.31) | 0.711 | 0.96 | 0.75 - 1.21 |
| rs2289195 | CONTROLS | 145(0.49) | 115(0.39) | 37(0.12) |  | 406(0.68) | 190(0.32) |  |  |  |
|  |  | **C/C** | **C/T** | **T/T** |  | **C** | **T** |  |  |  |
| DNMT3A | PATIENTS | 73(0.25) | 155(0.52) | 68(0.23) | 0.594 | 301(0.51) | 291(0.49) | 0.437 | 1.1 | 0.87 - 1.38 |
| rs734693 | CONTROLS | 82(0.28) | 143(0.49) | 64(0.22) |  | 307(0.53) | 271(0.47) |  |  |  |
|  |  | **G/G** | **GT** | **T/T** |  | **G** | **T** |  |  |  |
| DNMT3B | PATIENTS | 151(0.46) | 125(0.38) | 49(0.15) | 0.395 | 427(0.66) | 223(0.34) | 0.524 | 1.08 | 0.85 - 1.37 |
| rs1569686 | CONTROLS | 136(0.46) | 123(0.42) | 34(0.12) |  | 395(0.67) | 191(0.33) |  |  |  |
|  |  | **C/C** | **C/T** | **T/T** |  | **C** | **T** |  |  |  |
| DNMT3B | PATIENTS | 20(0.06) | 129(0.40) | 170(0.53) | 0.627 | 169(0.26) | 469(0.74) | 0.599 | 0.93 | 0.72 - 1.21 |
| rs2424913 | CONTROLS | 20(0.07) | 107(0.37) | 165(0.57) |  | 147(0.25) | 437(0.75) |  |  |  |
|  |  | **G/G** | **A/G** | **A/A** |  | **G** | **A** |  |  |  |
| DNMT3B | PATIENTS | 201(0.66) | 89(0.29) | 16(0.05) | 0.193 | 491(0.80) | 121(0.20) | 0.092 | 0.79 | 0.60 - 1.04 |
| rs2424932 | CONTROLS | 163(0.58) | 99(0.35) | 17(0.06) |  | 425(0.76) | 133(0.24) |  |  |  |
|  |  | **A/A** | **A/G** | **G/G** |  | **A** | **G** |  |  |  |
| DNMT3L | PATIENTS | 119(0.38) | 145(0.47) | 47(0.15) | 0.377 | 383(0.62) | 239(0.38) | 0.250 | 0.87 | 0.69 - 1.10 |
| rs8129776 | CONTROLS | 102(0.36) | 126(0.45) | 55(0.19) |  | 330(0.58) | 236(0.42) |  |  |  |
|  |  | **A/A** | **A/G** | **G/G** |  | **A** | **G** |  |  |  |
| DNMT3L | PATIENTS | 167(0.55) | 117(0.39) | 17(0.06) | 0.484 | 451(0.75) | 151(0.25) | 0.435 | 1.11 | 0.85 - 1.46 |
| rs762424 | CONTROLS | 167(0.60) | 95(0.34) | 17(0.06) |  | 429(0.77) | 129(0.23) |  |  |  |
|  |  | **C/C** | **C/T** | **T/T** |  | **C** | **T** |  |  |  |
| DNMT3L | PATIENTS | 183(0.60) | 102(0.34) | 18(0.06) | 0.177 | 468(0.77) | 138(0.23) | 0.060 | 0.77 | 0.59 - 1.01 |
| rs2070565 | CONTROLS | 143(0.53) | 105(0.39) | 22(0.08) |  | 391(0.72) | 149(0.28) |  |  |  |
|  |  | **C/C** | **C/T** | **T/T** |  | **C** | **T** |  |  |  |
| DNMT3L | PATIENTS | 222(0.71) | 82(0.26) | 7(0.02) | 0.611 | 526(0.85) | 96(0.15) | 0.419 | 1.15 | 0.82 - 1.60 |
| rs2838535 | CONTROLS | 191(0.75) | 58(0.23) | 6(0.02) |  | 440(0.86) | 70(0.14) |  |  |  |
